# Topsy-Turvy Heart with Aortopulmonary Window and Severe Airway Malacia: Prenatal Diagnosis and Review of the Literature

**DOI:** 10.1007/s00246-021-02710-1

**Published:** 2021-08-27

**Authors:** Yahia Hejazi, Gurdeep Mann, Younes Boudjemline, Jai P. Udassi, Grace Van Leeuwen, Karim A. Diab

**Affiliations:** 1 Division of Cardiology, Department of Pediatrics, Sidra Medicine, Doha, Qatar; 2 Division of Cardiac Imaging, Body Imaging Unit, Sidra Medicine, Doha, Qatar; 3 Division of Cardiac Intensive Care Unit, Department of Pediatrics, Sidra Medicine, Doha, Qatar

**Keywords:** Topsy-turvy heart, Superior–inferior ventricle, Aortopulmonary window, Vascular anomalies

## Abstract

The topsy-turvy heart is a very rare cardiac malformation that involves a global 90° clockwise rotation of the heart along its long axis. This rotation results in the displacement of the great arteries and severe elongation and stretching of the brachiocephalic arteries and the bronchi. We present an unusual case of topsy-turvy heart diagnosed prenatally with a large aorto-pulmonary window and. This case gives an insight into the morphological details and clinical presentation of this rare malformation and its associated complications. We also present a review of the literature of this rare anomaly showing only 15 live cases that have been published with only three cases diagnosed prenatally.

## Introduction

Topsy-turvy heart is a very rare cardiac malposition anomaly first described by Freedom et al. [1]. It involves a global 90° clockwise rotation of the entire heart with its great vessels around its long axis resulting in superior–inferior or upstairs–downstairs position of the ventricles. The right ventricle (RV) becomes more superior in relation to the left ventricle (LV), while the great arteries become displaced inferiorly and posteriorly into the mediastinum. This spatial derangement results in significant elongation of the brachiocephalic vessels and significant stretching of the trachea and bronchi leading to various degrees of airway anomalies and compression. Associated cardiac anomalies mainly include aorto-pulmonary (AP) window and atrial septal defects (ASDs). Its natural history and management are unclear due to the paucity of cases described in the literature to date.

We report a case of topsy-turvy heart prenatally diagnosed with AP window, suspected aortic coarctation, and lung deformities. We also summarize the cases reported in the literature of this rare anomaly, which include only fifteen living cases with only three cases diagnosed prenatally.

## Case Presentation

**Fig. 1:**
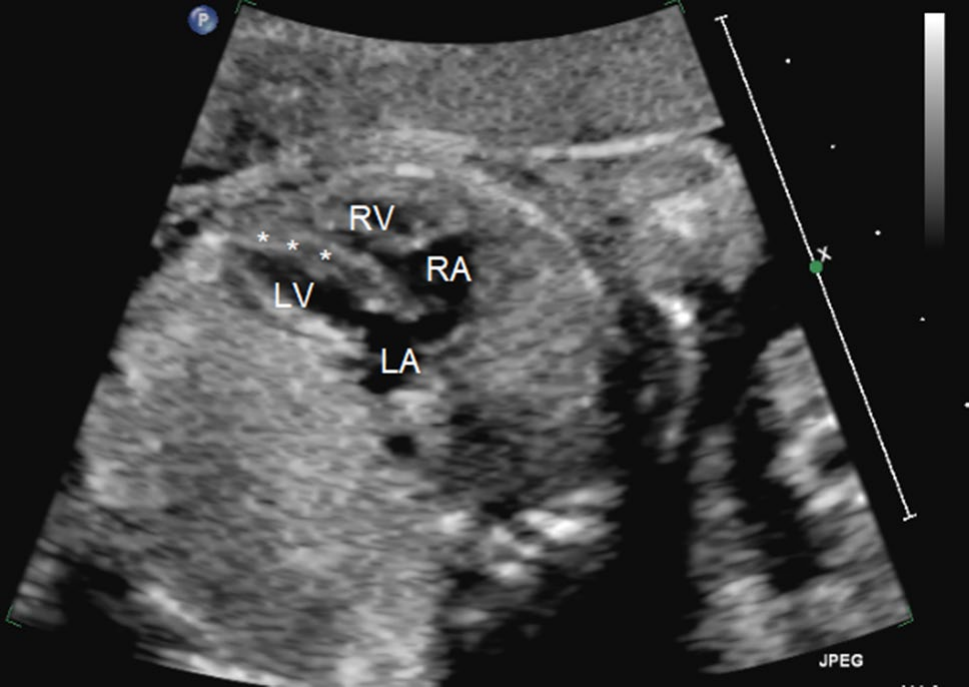
Fetal echocardiographic four-chamber view showing the abnormal horizontal position of the interventricular septum (*) as a result of the superior inferior rotation of the heart. *LV* left ventricle, *RV* right ventricle, *LA* left atrium, *RA* right atrium

**Fig. 2:**
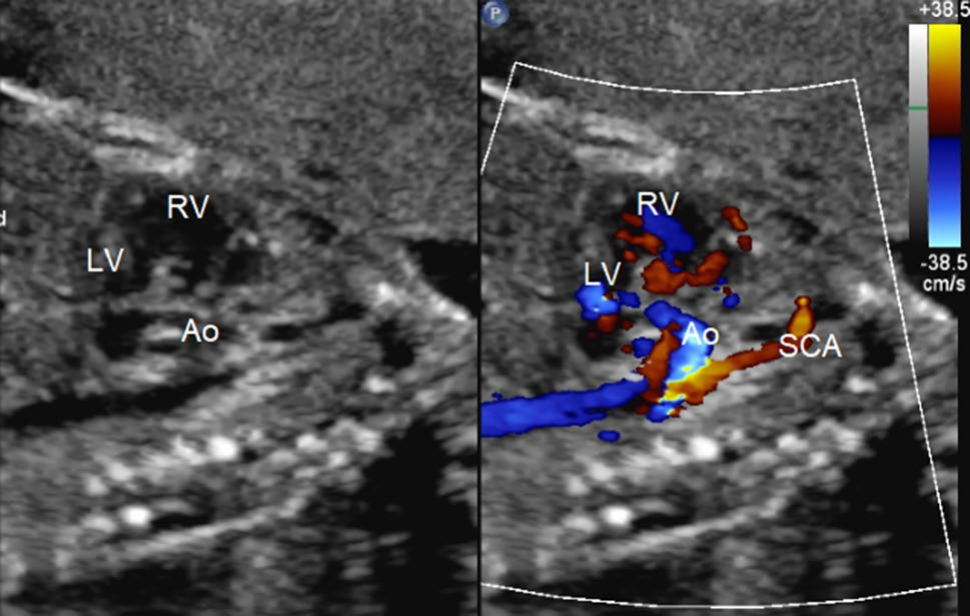
Fetal long axis echocardiographic view with 2D and color showing the superior–inferior relationship of the ventricles, the short ascending aorta, and an elongated subclavian artery. *LV* left ventricle, *RV* right ventricle, *Ao* aorta, *SCA* subclavian artery

**Fig. 3:**
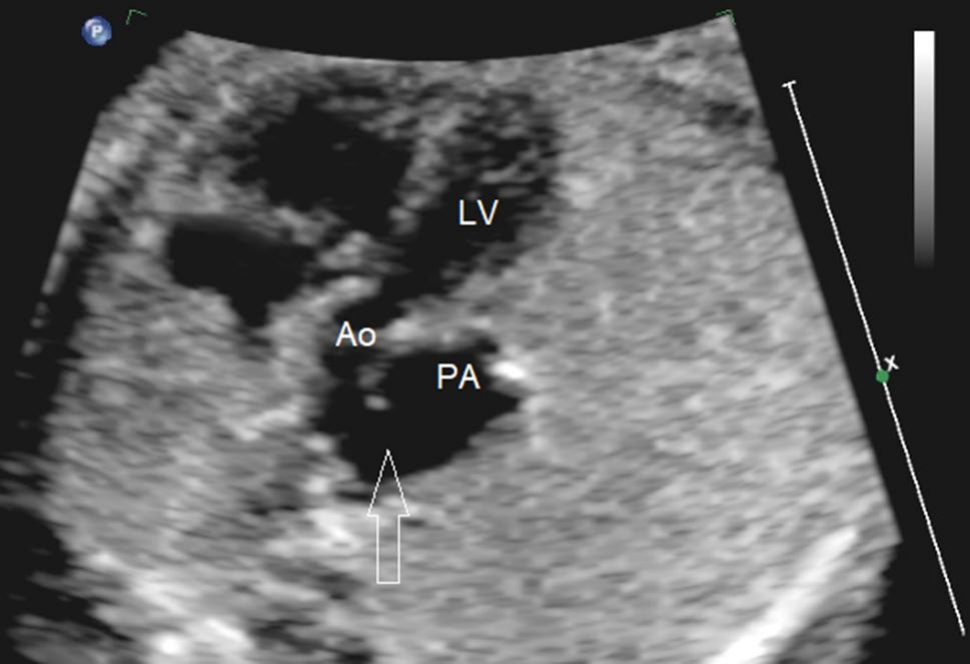
Fetal echocardiographic view showing the left outflow tract and a large AP window (arrow) connecting the aorta with the pulmonary artery. *Ao* aorta, *PA* pulmonary artery, *LV* left ventricle

A 20-year-old mother gravida 1 para 0 (G1P0) with history of congenital lobar emphysema was referred for fetal cardiac evaluation at 22 weeks of gestation because of suspected fetal cardiac anomaly with ventricular septal defect. Parents were first cousins with a family history of congenital bronchiectasis in the mother’s uncle and congenital cardiac abnormality (single ventricle physiology) in a paternal uncle. Fetal scan also revealed possible bronchial compression, single umbilical artery type 2 with persistent vitelline artery, and hypoplastic abdominal aorta. Fetal echocardiogram revealed superior–inferior ventricles with normal atrioventricular and ventriculoarterial relationships, a drop-out in the membranous ventricular septum suggestive of a defect, abnormally short ascending aorta, elongated subclavian artery, and a large AP window (Figs. 1, 2, 3). Pregnancy was smooth and the patient delivered at term by elective cesarean section (C/S) due to her pulmonary emphysema.

The baby was born with a birth weight of 2750 g, Apgar scores were 1, 6, and 8 at 1, 5, and 10 min, respectively. The baby was intubated right after delivery and was admitted to the neonatal intensive care unit (NICU). Post-natal echocardiogram confirmed the presence of superior–inferior ventricles. There was normal atrioventricular and ventriculo-arterial connections, a small secundum ASD, and a large AP window. There was no ventricular septal defect. Cardiac valves were morphologically and functionally normal except for a bicuspid aortic valve with no significant stenosis or regurgitation. The aortic arch was very difficult to visualize adequately due to its unusual course and tortuosity. The pulmonary artery was significantly dilated, the abdominal aorta showed significant flow reversal secondary to the AP window.

**Fig. 4:**
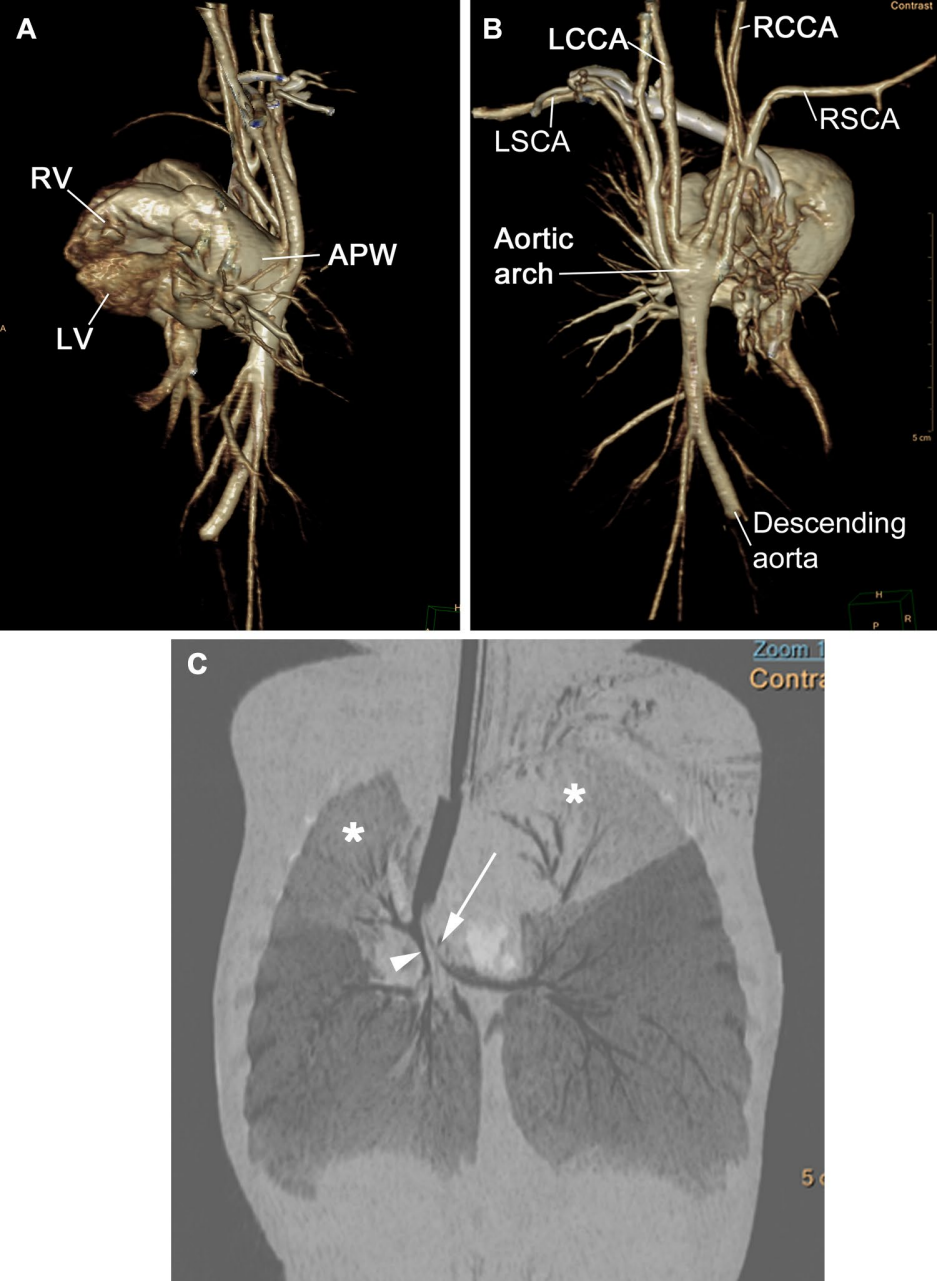
MDCTA reconstructed images. **A** Lateral 3DVR demonstrating superior RV and inferior LV with an APW. **B** Posterior 3DVR reveals four elongated head and neck arterial branches from the posteroinferior aortic arch and abrupt high termination of the abdominal descending aorta. **C** Coronal MinIP (lung window) reconstruction showing ribbon-like stretched right bronchus intermedius (arrowhead), collapsed proximal left main bronchus with bilateral upper lobe atelectasis (*), and ball-valve type hyperinflation of the remainder of the lungs. *MDCTA* multidetector CT angiogram, *3DVR* three-dimensional volume rendering, *APW* aortopulmonary window, *LCCA* left common carotid artery, *RCCA* right common carotid artery, *LSCA* left subclavian artery, *RSCA* right subclavian artery, *MinIP* minimum intensity projection, *superior RV and inferior LV* superinfero ventricles

Cardiac computed tomography (CT) showed upstairs–downstairs relationship of the ventricles, a distal non-restrictive AP window (Fig. 4). The head and neck vessels had an abnormal branching from a low short “pseudoarch” at the posterior aspect of the AP window. There was an abrupt termination of the abdominal aorta and the branches supplying the abdominal viscera and lower extremities were all gracile. Airway findings included a distal concentric tracheal stenosis with bronchus suis type airway to the right upper lobe. There was discrete narrowing of the distal trachea and near obliteration of the proximal main bronchi. The carina and proximal main bronchi were compressed as they pass between the right pulmonary artery and the distal main pulmonary artery-aortic continuation, with secondary areas of atelectasis. Having this rare and complex cardiac defect, consensus was to do cardiac cath to get more imaging to help understand the anatomy more precisely. Closing the AP window could have been also a reasonable ‘bridge’ management, allowing the patient to grow, which can help explore more extensive treatment options in the future.

**Fig. 5:**
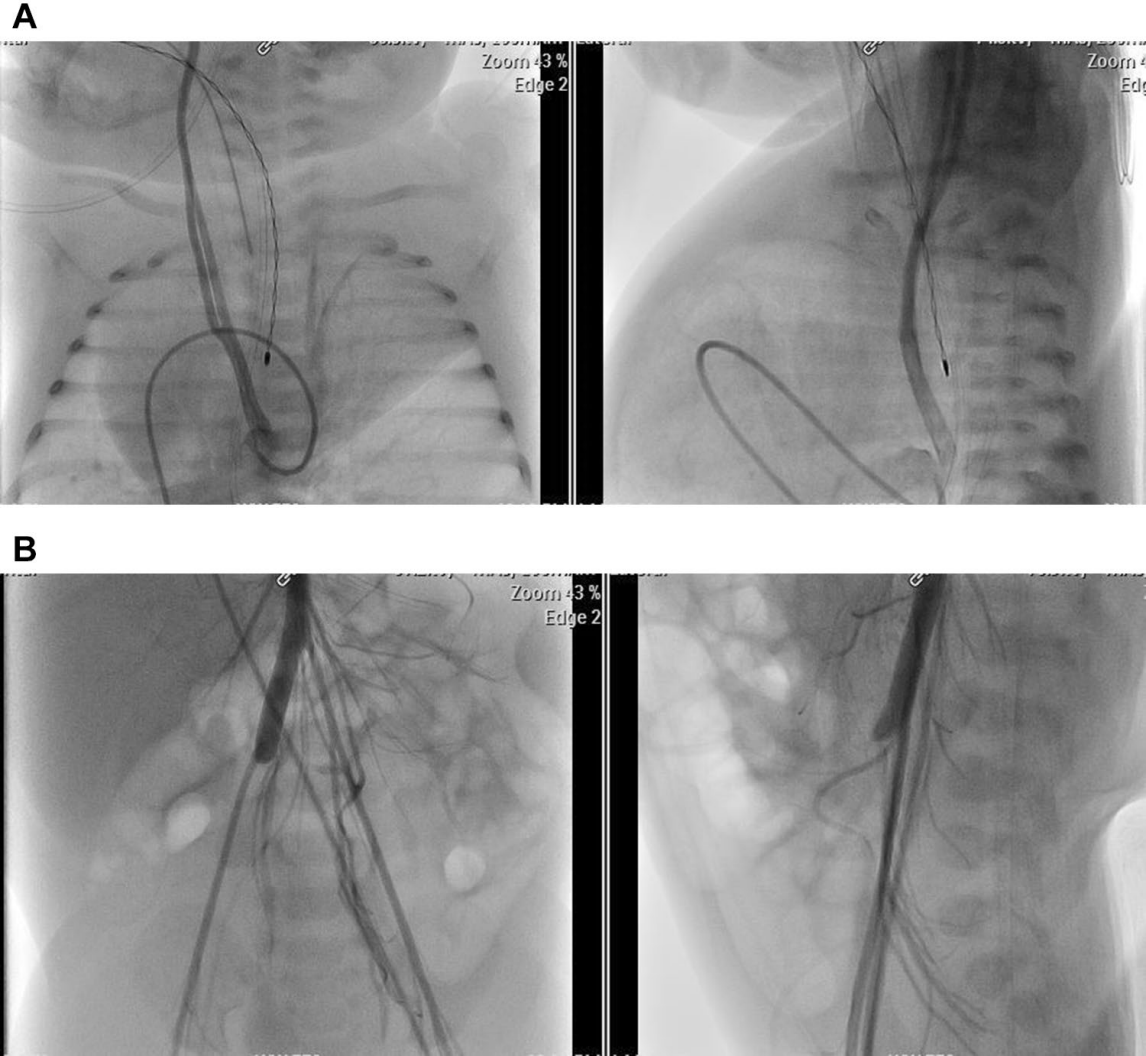
**A** Aortic angiogram with anteroposterior and lateral views: showing dextroposition, cardiac apex abnormally oriented to left upper thorax. Catheter course as following: inferior vena cava, right atrium, right ventricle, pulmonary artery, then through AP window to the aorta. Aortogram showing significant elongation of the head and neck vessels with abnormal take-off at level of diaphragm (contrast is filling only right common carotid artery in this image).** B** Aortogram, anteroposterior view, showing interruption of abdominal aorta, and multiple abnormal collateral vessels supplying the abdominal organs

Angiography showed dextrocardia, horizontally superimposed ventricles with the RV superior, and the LV posterior leading to the great vessels arising horizontally, low position of the great vessels in the thorax, and a large AP window. Because of the position and orientation of the great vessels, the position, branching, and direction of the pulmonary arteries were also unusual. Similarly, branching of the aorta was uncommon with neck vessels originating low from the descending aorta with a long vertical course in the chest to the arms, neck, and head. The abdominal aorta was interrupted with highly abnormal vascularization to the abdominal organs and legs through small vessels. Post-catheterization, the baby’s clinical status deteriorated with signs of decreased perfusion requiring vasopressor support and fluid resuscitation. A flexible bronchoscopy and bronchogram showed critical stenosis with malacia of left main bronchus with pulsatile compression noted and resultant ball-valve effect on the left lower lobe (Fig. 5).

A multidisciplinary team discussion led to a consensus that the airway lesion with extensive vascular airway compression and malacia were likely futile. Palliative care was offered and no intervention was performed. The baby was extubated to noninvasive ventilation and expired shortly afterward.

## Discussion

Topsy-turvy heart, first described in 1984 by Freedom et al. [1, 2], is characterized by superior–inferior spatial relationship of the atria and ventricles, and by downward displacement of the aortic arch and pulmonary trunk into the distal thorax with significant elongation of the brachiocephalic arteries. Abnormal cardiac malposition and ventricular relationships can be attributed to the following three mechanisms: twisting, tilting, and rotation [3].Twisting of the ventricular inlet components is the most common condition resulting in twisted atrioventricular connections and abnormal cardiac development. In this particular condition, cardiac chambers are arranged as if the heart was twisted clockwise (or anticlockwise) by a hand placed on the apex, causing a spiral relationship of the atria and ventricles, and resulting in a criss-cross heart. Ventricular tilting, on the other hand, is characterized by a superior–inferior relationship of the ventricles; as if the apex of the heart were simply lifted by a hand placed on the apex. Both of these conditions are associated with major intracardiac anomalies. The third, least common mechanism, involves a 90° clockwise rotation of the entire heart with its great vessels around its long axis, without any twisting of the atrioventricular connections resulting in the so-called topsy-turvy heart. This rotation results in the abnormal position of the brachiocephalic vessels, which become significantly elongated, and results in significant airway compression. Unlike the first two conditions, topsy-turvy heart is usually associated with only AP window without major intracardiac anomalies.

A review of the literature revealed a total of 17 previously reported cases with topsy-turvy heart, 3 of which were diagnosed prenatally (Table 1). Two of those pregnancies were terminated based on parents’ choice, resulting in 15 live cases. Most of the cases had normal intracardiac anatomy except AP window in eleven of the cases. Sasikumar et al. reported a case of topsy-turvy heart with AP window and ASD, for which the patient underwent AP window repair and ASD size reduction [4]. Alkhateeb et al. reported a case of topsy-turvy heart associated with inlet type ventricular septal defect in addition to AP window [5]. Bhalgat et al. reported the first case of topsy-turvy heart with criss-cross heart and AP window [6]. Öztürk et al. reported a 30 days male newborn with topsy-turvy heart associated with aortic arch interruption [7].

**Table 1:** Cases of topsy-turvy heart reported in the literature to date

References	Year of publication	Case #	Age at diagnosis	Associated cardiac defects	Gender	Pre/post-natal Dx	Consanguinity	Genetic test	Intervention and outcome
Freedom et al. [1, 2]	1984	1, 2, 3	NA	NA	NA	NA	NA	NA	NA
Jaeggi et al. [8]	2008	4	Fetus	None	M	Fetal at 19 weeks	First degree cousins	Normal karyotype, normal FISH test for 22q11.2 deletion or duplication	Termination as per parents willing and autopsy declined
		5	Fetus	None	F	Fetal at 18 weeks	First degree cousins	Normal karyotype, normal FISH test for 22q11.2 deletion or duplication	PDA ligation at age of 20 days. Suffered from air trapping, PHN crisis, prolonged hospital admission. At 11 months of age, underwent left tracheobronchial repair, aortic arch reconstruction, and flexible Genesis biliary stent was inserted into the LMB, achieving complete aeration of the left lung. Extubated and weaned gradually to room air. The patient has been home for nearly 2 years and is asymptomatic, thriving, and developmentally normal. A CXR follow-up has been satisfactory
Erek et al. [9]	2013	6	1 month	AP window	F	Postnatal	Yes	NA	AP window repair. Prominent air trapping in left lung. Severe respiratory failure. Passed away after six days of ECMO support due to multiorgan failure
		7	5 months	AP window	M	Postnatal	Yes	NA	AP window repair. Complicated with PHN crisis and VAP. Discharged home on the 30th postoperative days in good clinical condition. PA pressure was 30 mmHg after 3 months of discharge
		8	4 years	AP window	F	Postnatal	Yes	NA	Cardiac catheterization revealed an elevated PVR (12 WU), and pulmonary vasoreactivity test was considered unresponsive. Bosentan was started with plan for follow-up
Güzeltaş et al. [10]	2013	9	20 days	AP window	NA	Postnatal	NA	NA	NA
Sasikumar et al. [4]	2016	10	14 days	ASD, AP window	F	Postnatal	No	NA	AP window closure, ASD size reduction, discharged on POD 10
Bayramoglu et al. [11]	2017	11	2 days	AP window	F	Postnatal	Not mentioned	NA	AP window closure. Passed away three weeks post-surgery secondary to septicemia
Zakaria [12]	2019	12	5 months	AP window	F	Postnatal	Not mentioned	NA	Passed away, no surgical intervention was done
Öztürk et al. [7]	2020	13	30 days	Type A IAA, PDA	M	Postnatal	Not mentioned	NA	NA
		14	4 months	AP window	M	Postnatal	Not mentioned	NA	NA
		15	43 months	AP window	F	Postnatal	Not mentioned	NA	NA
Alkhateeb et al. [5]	2021	16	5 days	AP window, inlet VSD	M	Postnatal	Yes	NA	Decompensated heart failure, complicated chest problem of bronchial stenosis with right-sided pneumonia. The patient died of respiratory failure after less than 24 h of ventilation
Bhalgat et al. [6]	2021	17	Fetus	AP window, criss-cross heart	NA	Fetal at 22 weeks	No	NA	Termination as per parents willing and autopsy declined
Hejazi et al	2021	18	Fetus	AP window	F	Fetal at 22 weeks	First degree cousins	Normal chromosomal microarray analysis	Palliative care due to severe airway anomaly, baby passed away shortly after being weaned of respiratory support

*NA* not available, *AP* aortopulmonary, *ASD* atrial septal defect, *CXR* chest X ray, *FISH* fluorescent in situ hybridization, *ECMO* extra corporeal membrane oxygenation, *HLHS* hypoplastic left heart syndrome, *IAA* interrupted aortic arch, *LMB* left main bronchus, *PA* pulmonary artery, *PDA* patent ductus arteriosus, *PHN* pulmonary hypertension, *POD* post-operative day, *PVR* pulmonary vascular resistance, *VSD* ventricular septal defect, *VAP* ventilator associated pneumonia, *WU* Woods units

The genetic etiology in this rare malformation is still not clear. The cases reported in the literature are all of Middle Eastern or South Asian origin, which suggests a genetic background. In addition to our patient, six cases had consanguineous parents, while two did not and there was no available data for the remaining cases. The history of consanguinity supports a single-gene disorder with a recessive mode of inheritance. Genetic testing results were only reported by Jaeggi et al. and was normal (both karyotype and fluorescence in situ hybridization, FISH) [8]. Our patient also had normal chromosomes and microarray analysis.

In terms of outcome, 5 of the 15 live patients reported underwent surgical intervention. Four patients had AP window repair, two of which were discharged home and did fairly well based on the last follow-up reported [4, 9]. The other two patients did not survive post-AP window repair: one developed respiratory failure attributed to airway compression [9], and the other passed away 3 weeks post operatively secondary to septicemia [11]. The fifth patient underwent patent ductus arteriosus (PDA) ligation; however, the patient suffered from pulmonary hypertension crisis and had a prolonged hospital admission. He underwent complex airway and aortic arch reconstruction followed by left main bronchus stenting [13].

One patient did not require surgical intervention but required treatment for severe pulmonary hypertension [9]. Two patients did not undergo any intervention and passed away secondary to severe respiratory failure and decompensated heart failure [5, 12]. Our patient also expired after palliative care and agreement with the family. There were no data about treatment provided or outcome in the rest of the seven cases reported [1, 7, 10].

This review of this rare anomaly reveals significant complications in these patients including acute and chronic respiratory failure, recurrent lung infection, hyperactive airway and pulmonary hypertension, mostly secondary to complex airway compression. This requires immediate highly specialized medical and potential surgical care in addition to short- and long-term follow-up in these patients.

In conclusion, topsy-turvy heart is a rare congenital anomaly characterized by 90° clockwise rotation of the entire heart with its great vessels around its long axis without any twisting of the atrioventricular connections. Reviewing the literature and based on our case reported above, the management and prognosis are mainly dependent on the complexity of the vascular anomaly and extension of airway disease, both of which poses major challenges in planning any surgical intervention. Prenatal detection is significantly low for this rare anomaly. In addition to its rarity, this can also be attributed to the fact that the intracardiac anatomy is usually normal as discussed while the pathology affects mainly the extracardiac structures (arteries and airways). Having said that, abnormal orientation of interventricular septum along with AP window and abnormal aortic arch on fetal echocardiogram should raise concerns for possible topsy-turvy heart. A multidisciplinary team approach, including cardiology, cardiothoracic surgery, and pulmonology is needed to guide the management of such anomalies based on each individual center’s experience.

## References

[1] Freedom RM, Culham J, Moes C, Freedom RM, Culham J, Moes C (1984). Superoinferior ventricles: a consideration of so-called criss-cross atrioventricular connections. Angiography of congenital heart disease.

[2] Freedom RM, Mawson JB, Yoo SJ, Benson LN, Freedom RM, Mawson JB, Yoo SJ, Benson LN (1997). Twisted atrioventricular connections. So-called superoinferior ventricles or criss-cross heart. Congenital heart disease: textbook of angiography.

[3] Anderson RH, Yoo SJ, Anderson RH, Baker EJ, Penny D, Redington AN, Rigby ML (2010). Abnormal positions and relationship of the heart. Pediatric cardiology.

[4] Sasikumar D, Dharan BS, Menon S, Sivasubramanian S, Kapilamoorthy TR (2016). Surgical closure of aortopulmonary window in a topsy-turvy heart: a surgical challenge. Ann Thorac Surg.

[5] Alkhateeb A, Mansour A, Tanidir IC (2021). Topsy-turvy heart: a first recorded case report with intracardiac anomaly. Echocardiography.

[6] Bhalgat P, Shah J, Bhalgat P (2021). Antenatal assessment of topsy-turvy heart with crisscross inlet. J Am Coll Cardiol Case Rep.

[7] Öztürk E, Ergul Y, Güzeltaş A (2020). Electrocardiographic properties of topsy-turvy heart. J Electrocardiol.

[8] Jaeggi E, Chitayat D, Golding F, Kim P, Yoo SJ (2008). Prenatal diagnosis of topsy-turvy heart. Cardiol Young.

[9] Erek E, Guzeltas A, Ozturk NY, Kiyan G, Karakoc F, Akalin F, Odemis E, Arsan S (2013). Topsy-turvy heart: a very rare congenital rotational heart disease with tracheobronchial anomalies. World J Pediatr Congenit Heart Surg.

[10] Güzeltaş A, Öztürk E, Diker M (2013). Topsy-turvy heart: a very rare case of superoinferior ventricle. Pediatr Cardiol.

[11] Bayramoglu Z, Yılmaz R, Demir AA, Yekeler E, Dursun M, Dindar A, Nisli K, Omeroglu R (2017) Turvy heart and associated imaging findings. J Cardiovasc Comput Tomogr 11(5):417–418. ISSN 1934–5925. 10.1016/j.jcct.2017.04.008 10.1016/j.jcct.2017.04.008 28438441

[12] Zakaria RH (2019). Topsy-turvy heart: volume-rendered CT angiography. Radiology.

[13] Herrera P, Caldarone CA, Forte V, Holtby H, Cox P, Chiu P, Kim PC, Airway Reconstruction Team (2008). Topsy-turvy heart with associated congenital tracheobronchial stenosis and airway compression requiring surgical reconstruction. Ann Thorac Surg.

